# Supplementary material for: Mitochondrial Transcriptome Control and Intercompartment Cross-Talk During Plant Development
Source: Cells. 2019 Jun 13;8(6):583. doi: 10.3390/cells8060583 (PMC6627697; doi:10.3390/cells8060583)
Supplement: Supplementary file 1 [file cells-08-00583-s001.zip › Supplementary File 1.pdf]

Supplementary Data to:

Mitochondrial transcriptome control and inter-compartment cross-talk during plant development

Adnan Khan Niazi, Etienne Delannoy, Rana Khalid Iqbal, Daria Mileshina, Romain Val, Marta Gabryelska, Eliza Wyszko, Ludivine Soubigou-Taconnat, Maciej Szymanski, Jan Barciszewski, Frédérique Weber-Lotfi, José Manuel Gualberto and André Dietrich

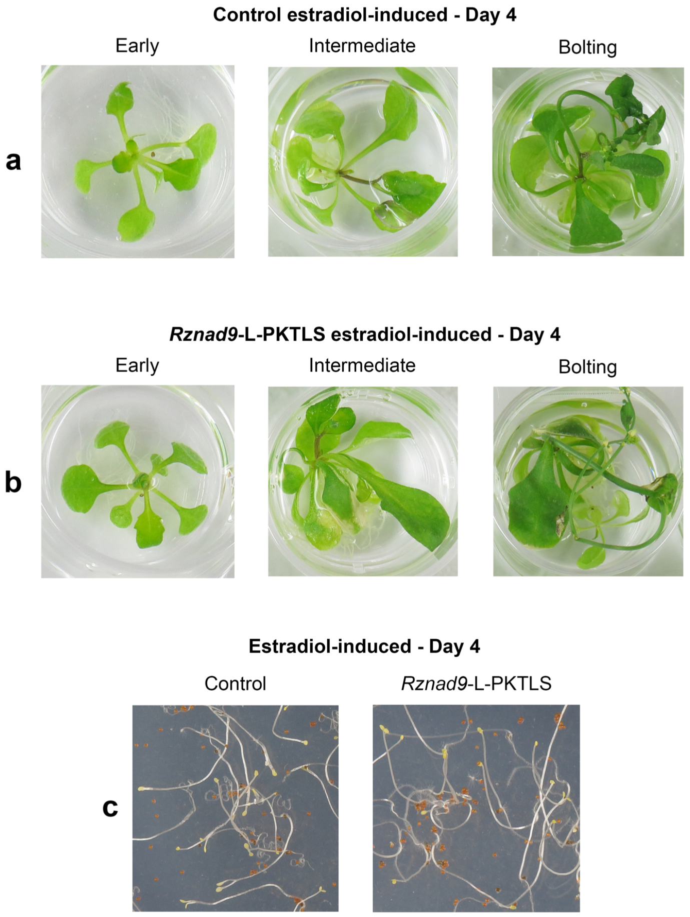

Figure S1. Phenotype of *A. thaliana* transgenic seedlings expressing the target-deprived *Rzsdh3* ribozyme (Control) or the *Rznad9* ribozyme (*Rznad9*-L-PKTLS). Seeds were germinated on Petri dishes containing solid MS-agar medium. Control (**a**) and *Rznad9*-L-PKTLS (**b**) seedlings were further grown in the light and transferred at the indicated growth stage (early, intermediate or bolting stage) to wells in culture plates containing liquid medium supplemented with estradiol. Pictures were taken at day 4 after the transfer to estradiol-supplemented medium, *i.e.* at the end of the time frame considered in our experiments. Alternatively, control and *Rznad9*-L-PKTLS seeds were germinated and grown on solid MS-agar medium under continuous dark and estradiol-supplemented liquid medium was added directly to the plates after 10 days of growth. Pictures were taken at day 4 after addition of estradiol-supplemented medium to the plates.

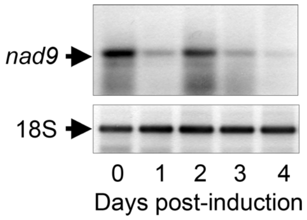

Figure S2. *Rznad9*-L-PKTLS-mediated knockdown of *nad9* steady-state levels in transformed *A. thaliana* seedlings at intermediate stage of growth. *A. thaliana* seeds carrying the *Rznad9*-L-PKTLS transgene were germinated in the light on solid MS-agar medium. Plants at intermediate stage of development (up to 10 true leaves) were transferred at Day 0 to wells in culture plates containing liquid medium supplemented with estradiol for transgene induction and samples were taken every day until Day 4 post-induction. A Day 0 to Day 4 northern blotting analysis of the steady-state level of the mitochondrial *nad9* target RNA is shown.

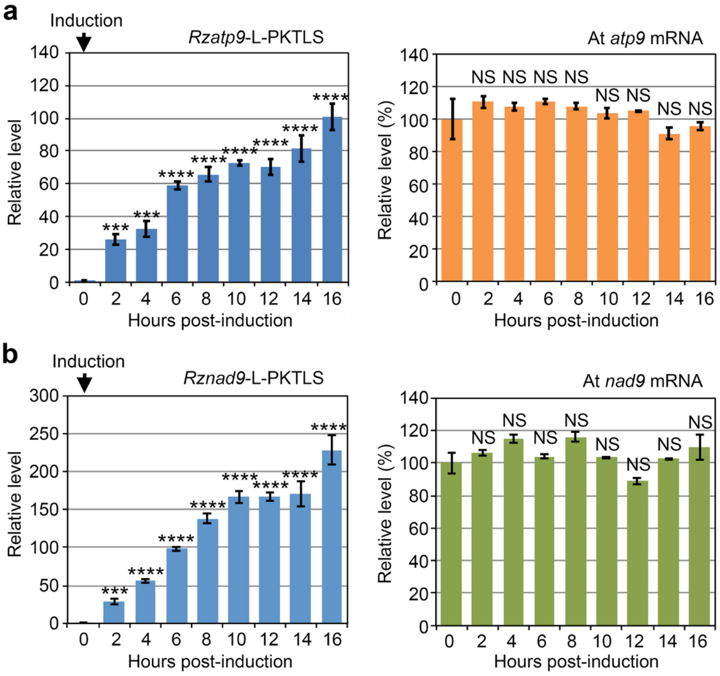

Figure S3. Short time kinetics of ribozyme expression and target RNA steady state level in *A. thaliana* plants at the early stage of development: no knockdown. *A. thaliana* seeds carrying the *Rzatp9*-L-PKTLS **(a)** or *Rznad9*-L-PKTLS (**b**) transgene were germinated in the light on solid MS-agar medium. Plants at early stage of development (maximum 4 true leaves) were transferred at time 0 to wells in culture plates containing liquid medium supplemented with estradiol for transgene induction and samples were subsequently taken every 2 hours. (**a**) Short time kinetics of induced expression of the *Rzatp9*-L-PKTLS RNA and of the steady-state level of the mitochondrial *atp9* target RNA as analyzed by RT-qPCR with total RNA from transformed plant samples. (**b**) Short time kinetics of induced expression of the *Rznad9*-L-PKTLS RNA and of the steady state level of the mitochondrial *nad9* target RNA. Data were analyzed with the Student's *t*-test; NS=not significant; ∗=p≤0.05; ∗∗=p≤0.01; ∗∗∗=p≤0.001; ∗∗∗∗=p≤0.0001.

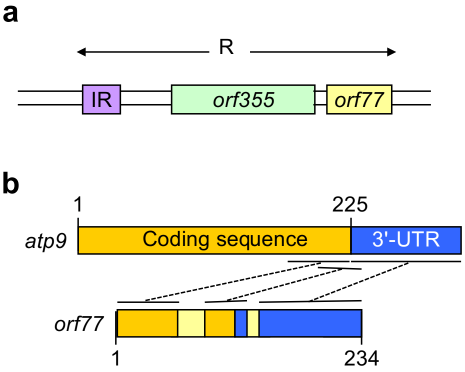

Figure S4. Common regions between the *orf77* and *atp9* sequences. (**a**) The mitochondrial genome of CMS-S maize contains a 4.2 kb repeat (R) that carries a small inverted repeat (IR) and two open reading frames (*orf355* and *orf77*). (**b**) The *orf77* is composed of sequences of unknown origin (pale yellow) and sequences shared with the coding (orange) and downstream (blue) regions of the mitochondrial *atp9* gene.

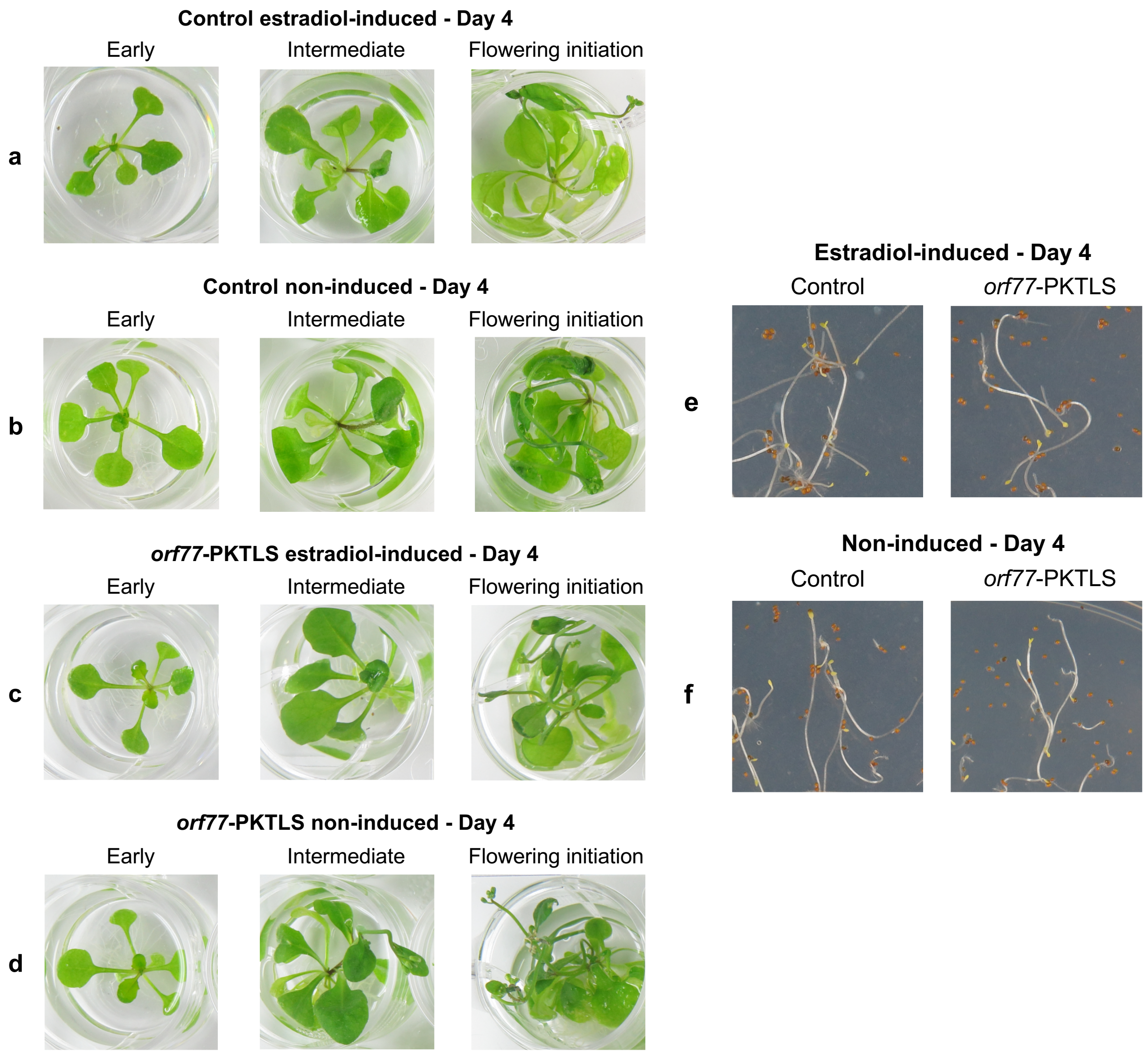

Figure S5. Phenotype of *A. thaliana* transgenic seedlings expressing the *orf77*-PKTLS RNA *versus* control seedlings. (**a**, **b**, **c**, **d**) Seeds were germinated on Petri dishes containing solid MS-agar medium. Seedlings were grown in the light and transferred at the appropriate developmental stage (early, intermediate or flowering initiation stage) to wells in culture plates containing either liquid medium supplemented with estradiol (estradiol-induced) or regular culture medium (non-induced), still in the light. The pictures were taken at day 4 after induction and show that neither the expression of the *orf77*-PKTLS RNA (panel **a** versus **c**), nor the estradiol treatment by itself (panel **a** versus **b**, panel **c** versus **d**) had a phenotypic effect within the 4-day time frame considered.(**e**, **f**) Seeds were sown on Petri dishes containing solid MS-agar medium that was overlayed after appropriate seedling growth in the dark with either liquid medium supplemented with estradiol (estradiol-induced) or regular culture medium (non-induced). The pictures were taken at day 4 after induction and show that neither the expression of the *orf77*-PKTLS RNA (panel **e**, left versus right), nor the estradiol treatment by itself (panel **e** versus **f**) had a phenotypic effect within the 4-day time frame considered.

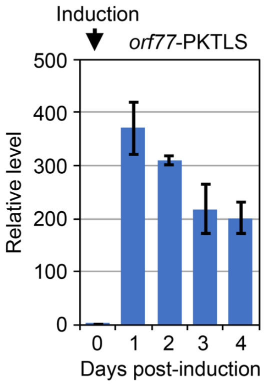

Figure S6. Kinetics of the *orf77*-PKTLS RNA expression at the early stage of development in light-grown transformed *A. thaliana* plants. *A. thaliana* seeds carrying the *orf77*-PKTLS transgene were germinated in the light on solid MS-agar medium. Plants at early stage of development (maximum 4 true leaves) were transferred at Day 0 to wells in culture plates containing liquid medium supplemented with estradiol for transgene induction and samples were subsequently taken every day until Day 4. Total RNA was extracted and analyzed by RT-qPCR.

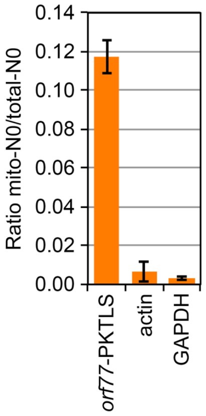

Figure S7. The *orf77*-PKTLS RNA is recovered in the mitochondrial fraction. Total RNAs and RNAs from purified mitochondria were prepared from light-grown transformed *A. thaliana* plants carrying the *orf77*-PKTLS transgene harvested at Day 2 after induction with estradiol. Total and mitochondrial RNAs were probed by RT-qPCR for *orf77*-PKTLS, actin and GAPDH. The results are given as the mitochondrial-N0 / total-N0 ratio.

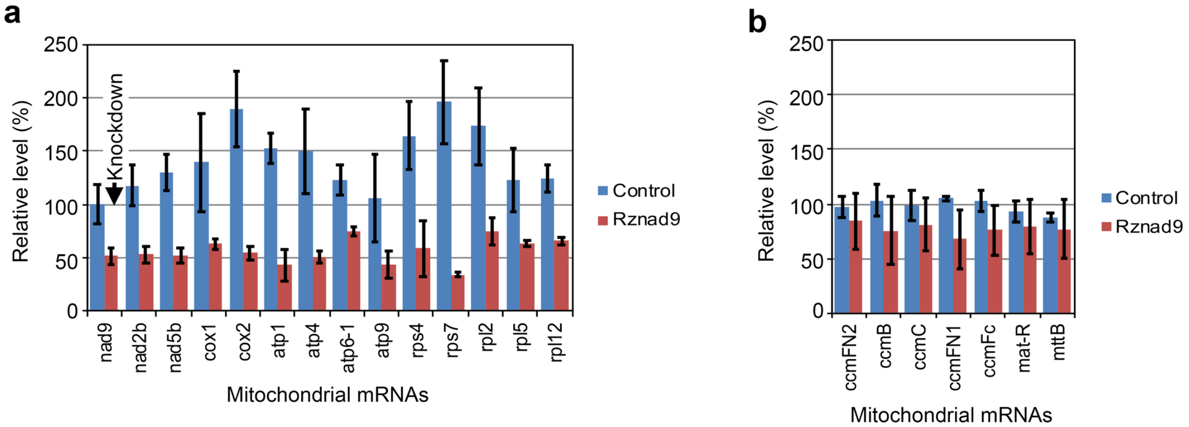

Figure S8. Knockdown of *nad9* negatively impacts the level of further mitochondrial mRNAs coding for subunits of oxidative phosphorylation complexes.Total RNAs were prepared from dark-grown transformed *A. thaliana* seedlings carrying the *Rzsdh3*-L-PKTLS transgene (Control, no target in *A. thaliana*) or the *Rznad9*-L-PKTLS transgene (Rznad9). Seedlings were harvested at Day 2 after induction of transgene expression with estradiol. The RNA fractions were probed by RT-qPCR for essentially all mitochondrial transcripts. For control samples, steady-state RNA levels are relative to Day 0 before estradiol induction. For *Rznad9* samples, steady-state RNA levels are relative to control. As indicated by an arrow, *nad9* decrease corresponds to specific *Rznad9*-L-PKTLS-mediated knockdown. A series of mitochondrial mRNAs coding for subunits of oxidative phosphorylation complexes or for ribosomal proteins showed an important decrease in relation with *nad9* knockdown (**a**), while mRNAs encoding biogenesis co-factors were little affected (**b**).
